# ICTV Virus Taxonomy Profile: *Geminiviridae* 2021

**DOI:** 10.1099/jgv.0.001696

**Published:** 2021-12-17

**Authors:** Elvira Fiallo-Olivé, Jean-Michel Lett, Darren P. Martin, Philippe Roumagnac, Arvind Varsani, F. Murilo Zerbini, Jesús Navas-Castillo, ICTV Report Consortium

**Affiliations:** ^1^​ Instituto de Hortofruticultura Subtropical y Mediterránea ‘La Mayora’, Consejo Superior de Investigaciones Científicas (IHSM-UMA-CSIC), 29750 Algarrobo-Costa, Málaga, Spain; ^2^​ CIRAD, UMR PVBMT, F-97410 Saint-Pierre, La Réunion, France; ^3^​ Computational Biology Group, University of Cape Town, 7925 Cape Town, South Africa; ^4^​ CIRAD, UMR PHIM, 34090 Montpellier, France; ^5^​ The Biodesign Center for Fundamental and Applied Microbiomics, Center for Evolution and Medicine, School of Life Sciences, Arizona State University, Tempe, AZ 85287, USA; ^6^​ Departamento de Fitopatologia, Universidade Federal de Viçosa, Viçosa 36570-900, MG, Brazil

**Keywords:** *Geminiviridae*, ICTV Report, taxonomy

## Abstract

The family *Geminiviridae* includes viruses with mono- or bipartite single-stranded, circular DNA genomes of 2.5–5.2 kb. They cause economically important diseases in most tropical and subtropical regions of the world. Geminiviruses infect dicot and monocot plants and are transmitted by insect vectors. DNA satellites are associated with some geminiviruses. This is a summary of the International Committee on Taxonomy of Viruses (ICTV) Report on the family *Geminiviridae* which is available at ictv.global/report/geminiviridae.

## Virion

**Fig. 1.**
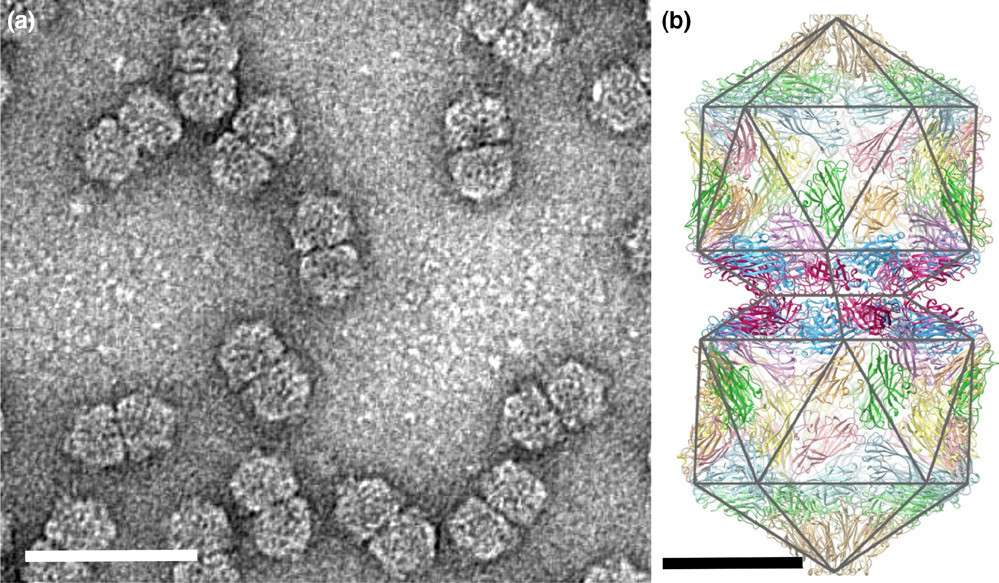
(**a**) Purified particles of maize streak virus stained with uranyl acetate showing typical twinned quasi-isometric subunits. Bar, 50 nm. Reproduced with permission from [1]. (**b**) Complete atomic model for all 110 subunits in the capsid of Ageratum yellow vein virus, with a polyhedral cage showing the symmetry of the particle. The fivefold symmetry axis is vertical and through the centre of the particle in this view. Bar, 10 nm. CC BY 4.0 licence from [2].

Geminiviruses have a unique particle morphology of twinned (geminate) icosahedra. For maize streak virus (genus *Mastrevirus*), virions are 22×38 nm, consisting of two incomplete icosahedra (*T*=1) containing 110 capsid protein (CP) subunits organized as 22 pentameric capsomers (Table 1, Fig. 1a) [1]. The structure of Ageratum yellow vein virus (genus *Begomovirus*) at 3.3 Å resolution shows that the N-terminus of CP adopts three different conformations essential for building the interface between geminate halves (Fig. 1b) [2].

**Table 1.** Characteristics of members of the family *Geminiviridae*

Example:	bean golden yellow mosaic virus (DNA-A: L01635, DNA-B: L01636), species *Bean golden yellow mosaic virus*, genus *Begomovirus*
Virion	Twinned (geminate) incomplete icosahedra, *T*=1, 22×38 nm with a single coat protein
Genome	2.5–5.2 kb of single-stranded, circular DNA, mono- or bipartite
Replication	Complementary strand synthesized in the nucleus by host replication factors; double-stranded circular molecules serve as templates for both transcription and replication; replication employs a rolling-circle mechanism and also a recombination-dependent mechanism
Translation	From transcribed mRNAs; members of some genera use transcript splicing
Host range	Plants (monocots and dicots)
Taxonomy	Realm *Monodnaviria*, kingdom *Shotokuvirae*, phylum *Cressdnaviricota*, class *Repensiviricetes*, order *Geplafuvirales*: >10 genera, >500 species

## Genome

**Fig. 2.**
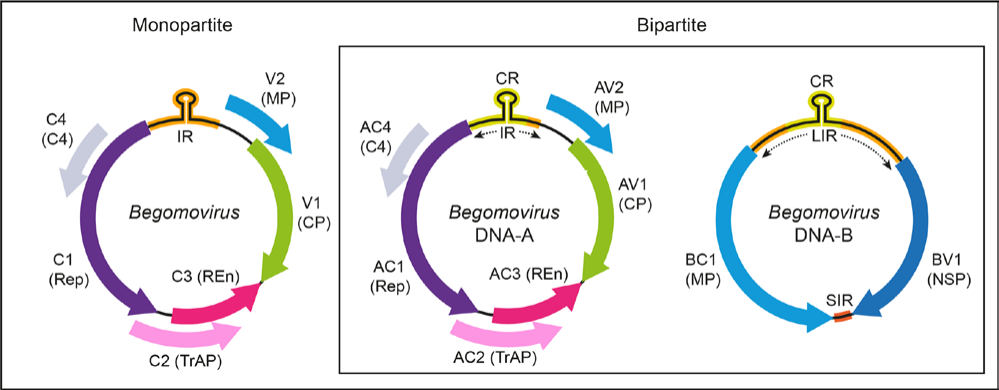
Genome organization of begomoviruses. The ORFs ([A]V1, [A]V2, [A]C1, [A]C2, [A]C3, [A]C4, BV1 and BC1) and their protein products (CP, capsid protein; MP, movement protein; Rep, replication-associated protein; TrAP, transcriptional activator protein; REn, replication enhancer protein; C4, C4 protein; NSP, nuclear shuttle protein) are shown. ORF [A]V2 is not present in New World begomoviruses. IR, intergenic region; LIR, long intergenic region; SIR, short intergenic region; CR, common region. The hairpin which includes the origin of replication is indicated in the IR/LIR.

Viruses in most genera of the family have monopartite genomes, whereas those in the genus *Begomovirus* have mono- or bipartite genomes. The genomes of bipartite begomoviruses (Fig. 2) consist of two circular single-stranded components, DNA-A and DNA-B, each of 2.5–2.6 kb. The two components share approximately 200 bases of sequence (common region) that includes the replication origin. DNA-A encodes a capsid protein (ORF AV1, CP), a putative movement protein (ORF AV2, MP, absent in New World begomoviruses), a replication-associated protein (ORF AC1, Rep), a transcriptional activator (ORF AC2, TrAP), a replication enhancer (ORF AC3, REn) and the C4 protein (ORF AC4, C4). DNA-B encodes a nuclear shuttling protein (ORF BV1, NSP) and a movement protein (ORF BC1, MP). The genomes of monopartite begomoviruses (Fig. 2) resemble the DNA-A component of bipartite begomoviruses [3, 4]. The genome organization of members of each of the genera in the family *Geminiviridae* is shown in the ICTV Report (ictv.global/report/geminiviridae). DNA satellites are associated with many begomoviruses and some mastreviruses [4].

## Replication

Complementary-sense DNA synthesis to produce dsDNA depends solely on host factors. Virus ssDNA synthesis is initiated by cleavage of the virion-sense strand by Rep in the 5′-TAATATTAC-3′ sequence conserved in most geminiviruses within the intergenic region (IR)/ long intergenic region (LIR). Geminiviruses do not encode a DNA polymerase, relying on host factors recruited during the early stages of replication. Coding regions in both strands diverge from the IR/LIR, and transcription is bi-directional. Geminiviruses use multiple overlapping transcripts for gene expression [3, 4]. The begomovirus tomato yellow leaf curl virus replicates in the salivary glands of whiteflies [5].

## Taxonomy

Current taxonomy: ictv.global/taxonomy. The family includes multiple genera collectively including >500 species, the most diverse genera being *Begomovirus* (>440 species) and *Mastrevirus* (>40 species). Host are dicots except for maldoviruses and mastreviruses (dicots and monocots) and eragroviruses (monocots). Vectors are whiteflies (begomoviruses), aphids (capulaviruses), treehoppers (grabloviruses, topocuviruses), leafhoppers (becurtoviruses, curtoviruses, mastreviruses, mulcrileviruses, turncurtoviruses), or unknown (citlodaviruses, eragroviruses, maldoviruses, opunviruses, topileviruses).

## Resources

Full ICTV Report on the family *Geminiviridae*: ictv.global/report/geminiviridae.
